# The effects of ginsenoside Rb1 on fatty acid β-oxidation, mediated by AMPK, in the failing heart

**DOI:** 10.22038/IJBMS.2018.24002.6016

**Published:** 2018-07

**Authors:** Hong-liang Kong, Ai-jie Hou, Ning-ning Liu, Bo-han Chen, Sheng-nan Dai, Hua-ting Huang

**Affiliations:** 1 Department of Cardiology, the People’s Hospital of Liaoning Province, i.e. the People’s Hospital of China Medical University, Shenyang, China; 2 Clinical Ophthalmology, the First Affiliated Hospital, China Medical University, Shenyang, China; 3 Dalian Medical University, Dalian, China

## Abstract

**Objective(s):**

This study intended to investigate the effects of *Ginsenoside-Rbl (Gs-Rbl)* on fatty acid β-oxidation (FAO) in rat failing heart and to identify potential mechanisms of *Gs-Rbl* improving heart failure (HF) by FAO pathway dependent on AMP-activated protein kinase (AMPK).

**Materials and Methods:**

Rats with chronic HF, induced by adriamycin (*Adr*), were randomly grouped into 7 groups. Gs-Rb1, adenine 9-β-D-arabinofuranoside (*Ara A*, specific AMPK inhibitor), and 5'-aminoimidazole-4-carboxamide riboside (*Aicar*, specific AMPK activator) were administered to rats with HF, singly and/or combinedly. Myocardial high-energy phosphate (such as phosphocreatine, ADP, and ATP), free L-Carnitine, malonyl-CoA, and the activity of FAO-related enzymes in left ventricle from different groups were measured by using the corresponding molecular biological techniques.

**Results:**

*Gs-Rb1* improved HF significantly, accompanied by a significant increase in phosphocreatine (PCr), ADP, ATP, PCr/ATP ratio, free carnitine, malonyl-CoA, mRNA, activity of carnitine palmitoyltransferase (Cpt), medium-chain Acyl-CoA Dehydrogenase (MCAD) and long-chain acyl-CoA Synthetase (ACSL) and a significant decrease of the ADP/ATP ratio in the left ventricular myocardium. However, all those effects were almost abolished by *Ara* A and were not further improved by *Aicar*.

**Conclusion:**

Taken together, it suggests that *Gs-Rb1* may modulate cardiac metabolic remodeling by improving myocardial fatty acid β-oxidation in failing heart. In addition, the effects of *Gs-Rb1 *may be mediated via activating AMPK.

## Introduction

Heart failure (HF) as a major health problem and a growing economic burden worldwide, is associated with abnormal myocardial energy metabolism (1, 2) and even provokes “metabolic remodeling” in the heart (3), characterized by a fetal metabolic phenotype (4, 5). “Metabolic remodeling” reduces the cardiac efficiency by converting chemical energy into mechanical work (6, 7) and stirs up abnormalities in myocardial substrate utilization from fatty acids to glucose (8). The decline in adenosine triphosphate (ATP) and the increase in free fatty acid concentration directly correlate with the progression of HF (8-12). As HF progresses towards an uncompensated state, metabolic adaptation becomes insufficient along with decreased mechanical efficiency (13). Therefore, improving “metabolic remodeling” has emerged as a promising approach for the treatment of HF.

Ginsenoside-Rbl (*Gs-Rbl*) as a major component of ginsenosides extracted from ginseng (the root of Panax ginseng C.A. MEYER, family Araliaceae) in Asian countries, has been revealed to ameliorate HF and protect the heart from ischemic and/or reperfusion injuries (14-20). Notably, our previous study showed that *Gs-Rb1* improves the viability of hypoxic cardiomyocytes by the regulation of glucose uptake, through the specific activation of glucose transporter-4 (14) and the enhancement of glycolysis, which were mediated by AMP-activated protein kinase (AMPK) (20). All of these indicate that *Gs-Rb1* may be a potential drug for ameliorating “metabolic remodeling” in the heart with HF. In HF, the lack of micronutrients may be an important reason that the heart cannot use the “fuel” (21). Carnitine is considered a “conditionally essential” nutrient, which decreases in chronic HF (21). Some studies showed that L-carnitine has the potential to improve HF (8, 21). However, the pharmacological effects of *Gs-Rb1* on myocardial fatty acid β-oxidation (FAO) in the failing heart are incompletely understood. In this study, we investigated the possible mechanisms of *Gs-Rb1* on mediating cardiac FAO. 

## Materials and Methods

All male Wistar rats (150-180 g) were obtained from the Laboratory Animal Center of China Medical University [SCXK (Liao) 2010-0001] and were housed singly fed with free access to food and water throughout the study. Animal care and experiments were conducted in accordance with the guidelines established by the Regulations for the Administration of Affairs Concerning Experimental Animals (Ministry of Science and Technology, China, revised in June 2004) and were approved by the People’s Hospital of Liaoning Province (i.e. The People’s Hospital of China Medical University). 

***Establishment of HF models and animal grouping***

Adriamycin (*Adr*, Sigma), *Gs-Rb1* (99.5%, the Research Center of Traditional Chinese Medicine, Wuhan, China), adenine 9-β-D-arabinofuranoside (*Ara A*; AMPK inhibitor; Gibco), and 5’-aminoimidazole-4-carboxamide riboside (*Aicar*; AMPK activator; Gibco) were freshly prepared. 

HF models were performed as we previously described (19, 22). Briefly, rats periodically received intraperitoneal (IP) injection of Adr at 0.2 mg/100 g five times over 3 days, followed by an additional five times over 1 week. At the 14^th^ day after final administration, HF was confirmed with echocardiographic examination and grouped randomly into groups as follows: control group (n=5), HF group (n=5), *Gs-Rb1* group (*Gs-Rb1* was administered in rat with HF, n=5), *Ara A-1* group (*Ara A* in rat with HF, n=5), *Ara A*-2 group (*Ara A* and *Gs-Rb1* in rat with HF, n=5), *Aicar*-1 group (*Aicar* in rat with HF, n=5), and *Aicar*-2 group (*Aicar* and *Gs-Rb1* in rat with HF, n=5). *Gs-Rb1* (10 mg/100 g body weight, dissolved in 1 ml normal saline), *Ara A* (50 mg/100 g, dissolved in 1 ml normal saline), and *Aicar* (50 mg/100 g, dissolved in 1 ml sterile saline) was administered IP daily for 7 days. Rats in the control group and in the HF group were administered 1 ml normal saline IP. Rats had available *ad libitum* a rat diet. After echocardiographic examination on the 8^th^ day, rats were humanely euthanized and blood and the left ventricle were collected. All tissues were snap frozen in liquid nitrogen and stored at -80 ^°^C for the following study.

***Echocardiographic examination***

Echocardiographic examination was performed by an investigator blinded to treatment allocation, according to our previously described method (19, 22). Briefly, after being anesthetized by IP injection of 10% chloral hydrate, two-dimensional and M-mode echocardiograms were obtained at the level of the papillary muscles with an echocardiographic system (CFM-725, 7.5M Hz broadband transducer, Vingmed, USA). Left ventricular dimensions were measured at least three consecutive cardiac cycles. Left ventricular end-diastolic volume (EDV), end-systolic volume (ESV), and left ventricular ejection fraction (LVEF) were acquired; LVEF= (EDV–ESV)/EDV×100%. The mean value of EF <0.45 was referred to as the standard of HF.

***Myocardial high-energy phosphate determination***

The high-energy phosphate substrate levels, including phosphocreatine (PCr), ADP and ATP, were measured by the HPLC-UV system (Shimadzu Corp, Kyoto, Japan) as previously reported (23). The left ventricular homogenate (final concentration 100 mg/ml), in 0.7 M ice-cold perchloric acid, was centrifuged at 15,000 g×5 min. The supernatant was neutralized for pH near 7.0 with 2 M potassium hydroxide and was then filtered through 0.45 µm filter and 10 µl was injected into a 3 μ Luna C-18 column using step gradient flow conditions. The mobile phase components, including 20 mM potassium phosphate buffer (pH 7.0) and 100% methanol, were delivered at a flow rate of 1 ml/min in the sequence of 100% phosphate buffer from 0–6.5 min, 100% methanol from 6.5–12.5 min followed by 100% phosphate buffer from 12.5 to 25 min for column re-equilibration in order to achieve stable baseline conditions. PCr, ADP and ATP were monitored at 210 nm. The standard curve range was from 6.25–100 µg/ml and the limit of detection was 0.078 µg/ml for ATP and 0.31 µg/ml for ADP. Intra- and inter-assay accuracy and precision ranged from 4.2% to 14.5%. PCr, ADP, and ATP contents were expressed as μmol/g tissue weight. 

***Free L-Carnitine analysis in the left ventricle***

Free L-carnitine in the left ventricle was quantified by HPLC-UV with pre-column derivatization as previously reported by researchers (23). Briefly, 50 mg pooled left ventricular samples were homogenized with 250 µl phosphate buffer (50 mM, pH 7.4) and were centrifuged at 2500 × g for 10 min at 4 ^°^C. Then 20 µl supernatant or serum sample was added to the reaction mixture and then incubated at 60 ^°^C for 2 hr followed by centrifugation at 12000 × g for 15 min depending on the kit. L-Carnitine was analyzed using a 10 µl sample with detection wavelength set at 260 nm.

***Determination of malonyl-CoA***

Malonyl-CoA levels were measured in freshly pre-pared left ventricular extracts using a rat malonyl-CoA ELISA kit (Sigma) according to the manufacturer’s protocol.

***Total mRNA isolation and quantitative RT-PCR analysis***

Total mRNA was extracted from different tissues using RNeasy Midi Kits according to the manufacturer of carnitine palmitoyltransferase (Cpt) kits’ instruc-tions. A poly-A tail was added to the extracted total RNA and was then reverse transcribed into cDNA to extend the RNA length. The expression of Cpt1b [primers (Forward/Reverse): cagccatgccaccaagatc/aagggccgcacagaatcc. accession number: NM_013200] and Cpt2 [primers (Forward/Reverse): gctccgaggcgtttctca/tggccgttgccagatagc. accession number: NM_012930] was examined by real-time qPCR using β-actin [primers (Forward/Reverse): agcgtggctacagcttcacc/tgccacaggattccataccc. accession number: NM_031144] as internal controls and was quantified following cDNA annealing using real-time PCR primers. 

***Assessment of heart Cpt enzyme activities***

The activities of the Cpt enzyme were measured using the spectrophotometric method as previously described (23). Briefly, frozen tissue was homogenized in 10% homogenization buffer supplemented with 3 mg nagarse and then centrifuged at 500 × g for 10 min at 4 ^°^C. The supernatant was centrifuged at 9000 × g for 35 min at 4 ^°^C. The pellet, being washed with the homogenization buffer without nagarse, was centrifuged at 9000 × g for 35 min at 4 ^°^C and resuspended in 200 µl isolation buffer without nagarse. Protein concentrations were measured using the Advanced Protein Assay kit (Sigma) with bovine serum albumin as standards. To determine total Cpt activity, 20 µg protein was assayed in 200 µl ml reaction buffer. Cpt2 activity was determined using the same reaction conditions as total Cpt without 10 µl malonyl-CoA (Cpt1 inhibitor, a final concentration of 10 μM). Cpt1 activity was calculated by subtracting the Cpt2 activity from the total Cpt activity. The Cpt activity was calculated as amount of CoASH released per min per mg protein. 

***Assessment of enzyme of medium chain acyl-CoA dehydrogenase (MCAD) and long-chain acyl-CoA synthetase (ACSL)***

The activities of MCAD and ACSL were measured as a marker of the capacity for fatty acid β-oxidation (FAO). MCAD activity was measured spectrophotometrically in mitochondria extracts, as previously described (24). ACSL specific activity was measured in mitochondrial homogenates (25). Briefly, 2 µg protein was incubated with 50 µM [1-^14^C]fatty acid, 250 μM CoA, 10 mM ATP, 5 mM dithiothreitol, and 8 mM MgCl_2_ in 175 mM Tris (pH 7.4) at room temperature for 10 min and then the enzyme reaction was stopped with 1 ml Dole’s solution. Radioactivity of the acyl-CoAs in the aqueous phase was measured using a liquid scintillation counter.

***Data analysis***

All data in this study were presented as mean ± standard error (`x±s). All experimental data were analyzed using PASW Statistics 22 (SPSS Inc, Chicago, USA). Multiple comparisons for all parameters at different groups were analyzed using one way ANOVA with Dunnett’s T3 test as *post hoc* test. *P*-values <0.05 were considered to be significant. 

## Results

**Figure 1:**
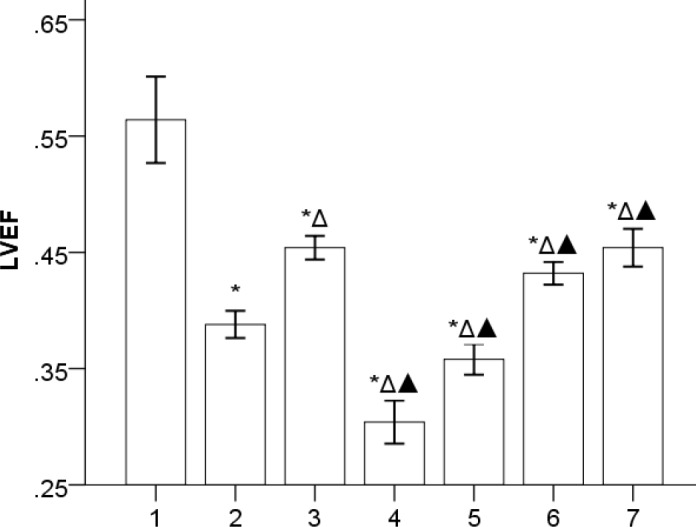
Comparison of LVEF value (`x±s, n=5): 1, 2, 3, 4, 5, 6, and 7 represent control, HF, *Gs-Rbl*, *ara A-*1, *ara A-*2*, Aicar-*1, and *Aicar-*2 groups, respectively. LVEF was 0.56±0.04 in control group; 0.39±0.01 in HF group; 0.45±0.01 in *Gs-Rb1* group; 0.30±0.02 in *ara A*-1 group; 0.36±0.01 in *ara A*-2 group; 0.43±0.01 in *Aicar*-1 group, and 0.45±0.02 in *Aicar*-2 group. *P<0.05 vs control group; Δ*P*<0.05 vs HF group; ▲*P*<0.05 vs *Gs-Rbl* group

***Gs-Rb1 improving cardiac functions of the rat chronic HF model (*** Figure 1 ***)***

Chronic HF rat models were successfully established by periodically injecting IP Adr. *Gs-Rb1* and *Aicar-1* significantly improved HF (*P*<0.05), however, the synergistic effect was not found between *Gs-Rb1* and *Aicar* (*P*>0.05). *Ara A* provoked HF to further deteriorate (*P*=0.000), which was improved by *Gs-Rb1* (*P*=0.027). 

**Figure 2:**
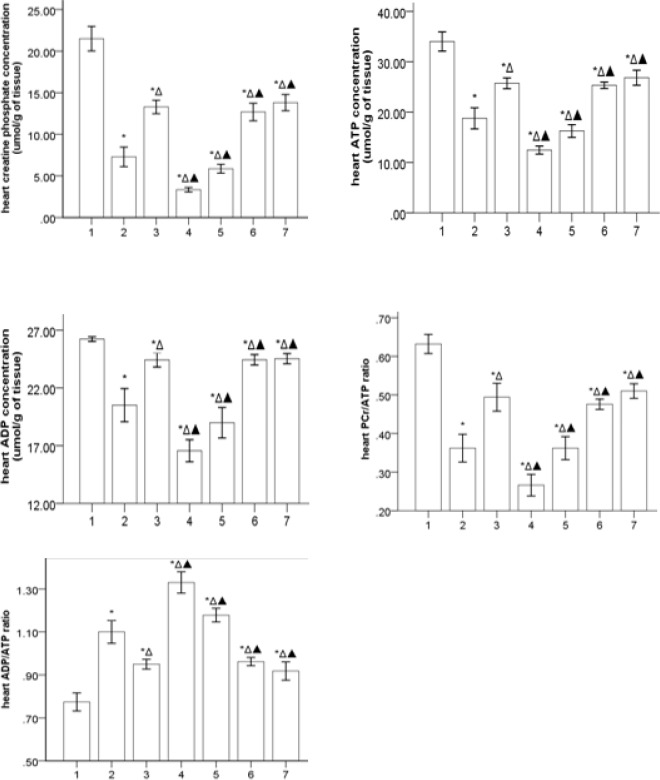
Heart high energy phosphate substrate profiles in different groups (`x±s, n=5) 1, 2, 3, 4, 5, 6, and 7 represent control, HF, *Gs-Rbl*, *ara A*-1, *ara A*-2, *Aicar*-1, and *Aicar*-2 groups, respectively. PCr/ATP ratio: there was significance in *ara A-*1 and *ara A-*2 groups (*P*=0.024), and there was no significance in *Gs-Rbl*, *Aicar*-1, and *Aicar*-2 groups. ADP/ATP ratio: the ratio was decreased in the *ara A*-2 group more than in the *ara A*-1 group (*P*=0.020), and there was no significance in *Gs-Rbl*, *Aicar*-1, and *Aicar*-2 groups. *P<0.05 vs control group; Δ*P*<0.05 vs HF group; ▲*P*<0.05 vs *Gs-Rbl* group

***Gs-Rb1 increasing the high energy phosphate substrate*** ***levels (***Figure 2***)***

*Gs-Rb1* and/or *Aicar* significantly increased each concentration of PCr, ATP, and ADP in rats with HF (*P*<0.01), in addition, *Gs-Rb1* significantly improved the inhibiting effects of *Ara A* on PCr, ATP, and ADP in rats with HF (*P*<0.01). However, there was no significance in *Gs-Rb1* and/or *Aicar* (*P*>0.05). Compared to the control group, HF groups significantly reduced the ratio between PCr and ATP (PCr/ATP ratio, *P*=0.000) and significantly increased the ratio between ADP and ATP (ADP/ATP ratio, *P*=0.000), which was further worsened by *Ara A* (*P*<0.05) and significantly improved by *Gs-Rb1*, *Aicar*, and *Aicar*+*Gs-Rb1* (*P*<0.05). However, no synergistic effect was found between *Gs-Rb1* and *Aicar* (*P*>0.05). 

**Figure 3:**
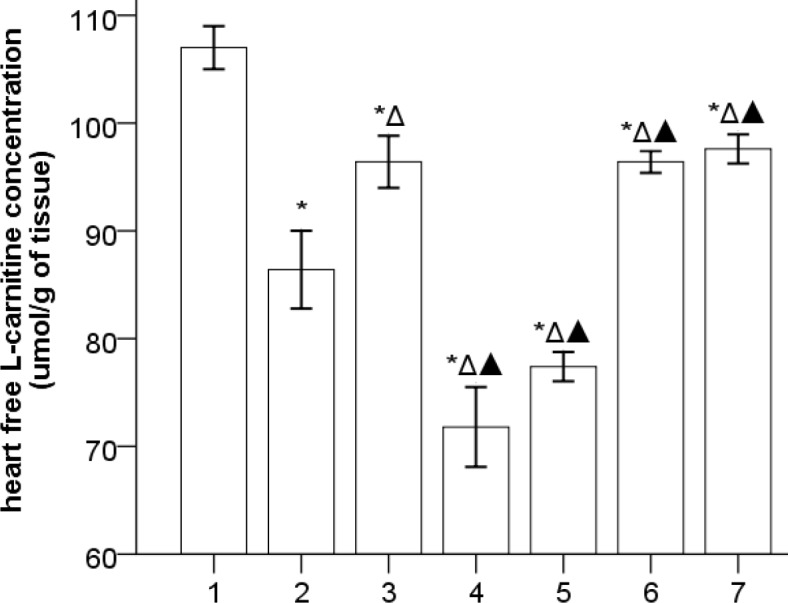
The levels of myocardial free L-carnitine (`x±s, n=5) 1, 2, 3, 4, 5, 6, and 7 represent the control, HF, *Gs-Rbl*, *ara A*-1, *ara A*-2, *Aicar*-1, and *Aicar*-2 groups, respectively. *P<0.05 vs control group; Δ*P*<0.05 vs HF group; ▲*P*<0.05 vs *Gs-Rbl* group

***The effects of Gs-Rb1 on free L-carnitine in the left ventricle (*** Figure 3 ***)***

The levels of myocardial free carnitine decreased in HF, which was further decreased by *Ara A* (*P*<0.01) and was significantly increased by *Gs-Rb1*, *Aica*r, and *Aicar*+*Gs-Rb1* (*P*<0.01). However, *Gs-Rb1* could not significantly change the effects of *Ara A* (*P*>0.05), and no differences were found between *Gs-Rb1*, *Aicar,* and *Aicar*+*Gs-Rb1* (*P*>0.05). 

**Figure 4:**
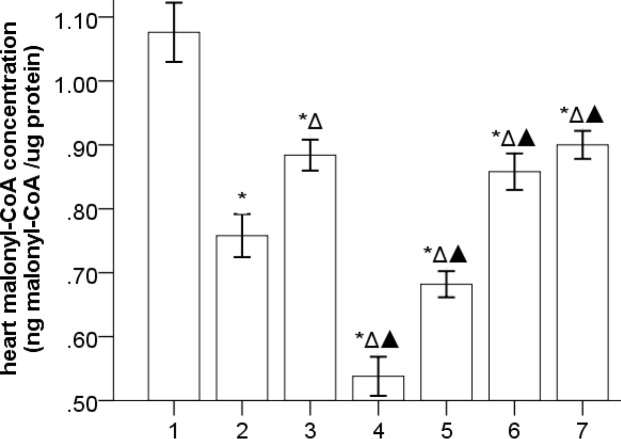
The levels of myocardial free L-carnitine (`x±s, n=5) 1, 2, 3, 4, 5, 6, and 7 represent control, HF, *Gs-Rbl*, *ara A*-1, *ara A*-2, *Aicar*-1, and *Aicar*-2 groups, respectively. *P<0.05 vs control group; Δ*P*<0.05 vs HF group; ▲*P*<0.05 vs *Gs-Rbl* group

***The effects of Gs-Rb1 on malonyl-CoA in the left ventricle (*** Figure 4 ***)***

The concentration of malonyl-CoA was significantly declined in the HF group compared to the control group (*P*=0.000), which was further deteriorated by *Ara A* (*P*=0.000) and significantly improved by *Gs-Rb1* (*P*=0.006), *Aicar* (*P*=0.030), and *Aicar*+*Gs-Rb1* (*P*=0.003), however, there existed no synergistic effect between *Gs-Rb1* and *Aicar* (*P*>0.05). In addition, *Gs-Rb1* significantly improved the inhibiting effect of *Ara A* on heart malonyl-CoA (*P*=0.002).

**Figure 5:**
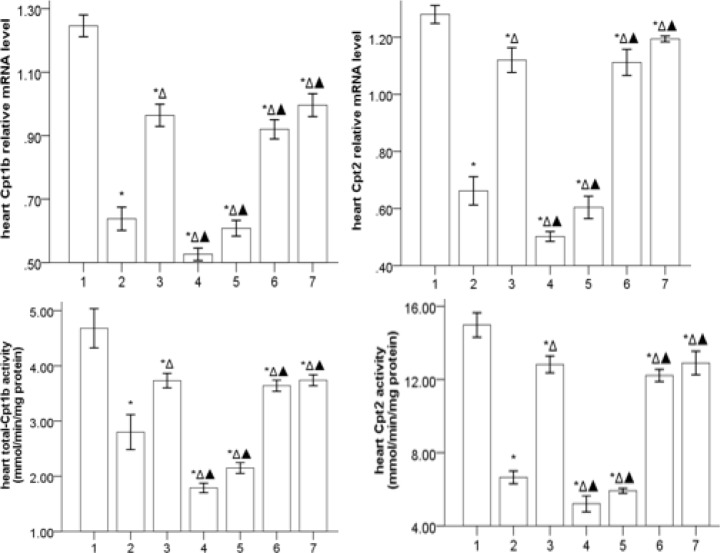
Cpt mRNA and Cpt activity levels in the myocardium (`x±s, n=5) 1, 2, 3, 4, 5, 6, and 7 represent control, HF, *Gs-Rbl, ara A*-1, *ara A*-2, *Aicar*-1, and *Aicar*-2 groups, respectively. *P<0.05 vs control group; Δ*P*<0.05 vs HF group; ▲*P*<0.05 vs *Gs-Rbl* group

***Gs-Rb1 ameliorating the expression of Cpt mRNA and Cpt activity (*** Figure 5 ***)***

The mRNA expression of Cpt1b and Cpt2 was significantly downregulated in HF (*P*<0.05) and reduced further by *Ara A* (*P*<0.05) and upregulated by *Gs-Rb1*, *Aicar,* and *Aicar*+*Gs-Rb1* (*P*<0.05) with no differences in the three groups (*P*>0.05). In addition, *Gs-Rb1* significantly improved the effects of *Ara A* (*P*<0.05). Most importantly, we also found that the activities of Cpt1b and Cpt2 were similar to the mRNA expressions of Cpt1b and Cpt2 in different groups.

**Figure 6:**
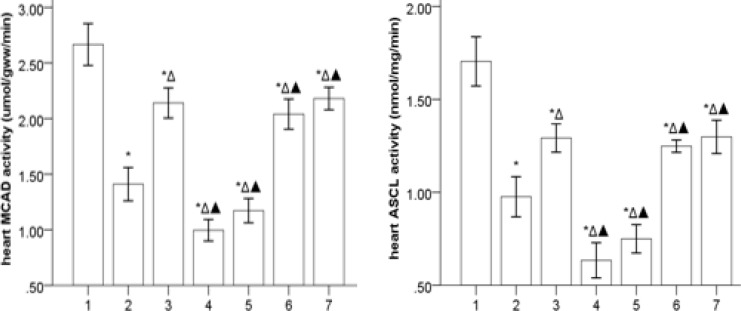
The activity of MCAD and ACSL on the failing heart (`x±s, n=5) 1, 2, 3, 4, 5, 6, and 7 represent control, HF, *Gs-Rbl, ara A*-1, *ara A*-2, *Aicar*-1, and *Aicar*-2 groups, respectively. *P<0.05 vs control group; Δ*P*<0.05 vs HF group; ▲*P*<0.05 vs *Gs-Rbl* group

***Gs-Rb1 modifying the activity of MCAD and ACSL in the failing heart (*** Figure 6 ***)***

The activities of MCAD and ACSL were significantly decreased in the HF group compared to the control group (*P*<0.05), which was further deteriorated by *Ara A* (*P*<0.05) and significantly improved by *Gs-Rb1*, *Aicar,* and *Aicar*+*Gs-Rb1* (*P*<0.05) without synergistic effects between *Gs-Rb1* and *Aicar* (*P*>0.05). In addition, *Gs-Rb1* did not significantly improve the effects of *Ara A* (*P*>0.05).

## Discussion

lot of unknown energy metabolic pathways might mediate the effects of *Gs-Rb1* such as inhibiting cell apoptosis, suppressing local inflammation, and improving glucose metabolism (14-20). Besides, the functional AMPK significantly contributes to restoration of myocardial contractile efficiency (26), which is one of the essential conditions for preserving cardiac function reported by Juric *et al* (27). In view of metabolic remodeling being integral to the progression of HF (10, 11) and *Gs-Rb1* improving glucose uptake and glycolysis (22), the purpose of the present study was to determine whether and how *Gs-Rb1* improves the FAO remodeling in the failing heart. In order to determine whether AMPK mediated the effects of *Gs-Rb1* on the failing heart, both *Ara A* (specific AMPK inhibitor) and *Aicar* (specific AMPK activator) were administrated. Our findings, consistent with prior studies (15), demonstrated that *Gs-Rb1* might improve Adr-induced HF (22). In addition, the findings, *Aicar* improving HF, *Ara A* deteriorating HF, the effect of *Gs-Rb1* improving HF being partly inhibited by *Ara A* and superior to *Aicar*, supported the above views reported by Juric* et al.* (27) and showed that *Gs-Rb1* possessed the effect of the AMPK activator and improved HF by activating the AMPK pathway.

Persistent pump function of the heart is supported by consuming quantities of ATP, about 60–90% of which is produced by the FAO in mitochondria. With pathologic cardiac remodeling in the failing heart, the heart also undergoes energy metabolic reprogramming: fuel substrate preferences shift from fatty acids to glucose and the capacity and efficiency of mitochondrial ATP production are diminished (8), being difficult to match with energy demands under diverse developmental and physiological circumstances. In a word, a failing heart has been referred to as “an engine out of fuel” (8). Both the reduction in the ratio of PCr/ATP, as an early sign of heart dysfunction and a predictor of mortality for patients with dilated cardiomyopathy (28), and the increase in the ratio of ADP/ATP have been associated with several pathological conditions (29). Consistent with the previous studies (9-12, 30-32), the present study further demonstrated that HF may provoke some obvious adverse changes of high-energy phosphates. All those adverse changes being deteriorated by *Ara A* and improved by *Aicar*, suggested further that the AMPK pathway takes part in the metabolism of myocardial high-energy phosphate (27). The present findings suggesting the effects of Gs-Rbl on myocardial high-energy phosphates were beyond the one by *Ara A* and similar to the one by *Aicar* in the failing heart, indicated that the AMPK pathway played a key role in mediating the effects of Gs-Rbl. 

L-Carnitine plays a key role in energy production as facilitates the transport of long-chain fatty acids across the mitochondrial membrane making them available for FAO (33, 34). Tissues with low L-carnitine levels often have low FAO rates (35) and typical Low CrP/ATP ratios (36). The reduction in L-carnitine levels may provoke the development of cardiomyopathy (21, 23, 37). The finding that heart free L-carnitine levels were reduced in HF groups, further showed the role of L-carnitine in HF (37). However, the reasons for out-of-balance L-Carnitine homeostasis were unknown. Malonyl-CoA levels, as endogenous inhibitors of Cpt1 and key regulators of myocardial substrate use (38), are the major determinants of the FAO rate (39, 40), which regulates long-chain fatty acyl CoA import into the mitochondria for FAO. AMPK, as an essential for controlling malonyl-CoA content (41), decreases malonyl-CoA production in the heart (41). Our data, the effects of *Ara A*/*Aicar* on L-Carnitine homeostasis, further demonstrated that AMPK played an important role in adjusting L-Carnitine homeostasis. The finding that the effects of *Gs-Rb1* on improving L-Carnitine and malonyl-CoA in the failing heart being inhibited by *Ara A* and similar to *Aicar*, demonstrated that *Gs-Rb1* administration may alter the homeostasis of both L-carnitine and malonyl-CoA in rats with HF, which were dependent on the AMPK pathway. Of particular note was that there exist other pathways mediating the effects of *Gs-Rb1* besides AMPK pathway.

Cpt, as a key transporter for long-chain fatty acids into the mitochondrial matrix space, consists of Cpt1 and Cpt2, of which Cpt1 is responsible for the first rate-limiting step in the FAO in mitochondria and its activation is most consistently associated with glutathiolation of Cpt1b. FAO is impaired and the activity of Cpt1 is markedly decreased in HF (24, 42-46), in which the impaired FAO is associated with concomitant decreases in the activity of Cpt1 (47). Both MCAD and ACSL are key enzymes of mitochondrial FAO, which plays a pivotal role in maintaining body energy homeostasis mainly during catabolic states. Consistent with the previous investigations (24, 42-46, 48, 49), MCAD activity, together with ACSL activity, was reduced in the failing heart, which demonstrated that the impaired FAO was associated with concomitant decreases in the activity and protein expression of MCAD. Our results indicating down-regulation of both mRNA and activity for Cpt1b and Cpt2 in HF are consistent with many previous studies (24, 42-46). The present study also demonstrated that the influence of *Gs-Rb1* on Cpt1b, Cpt2, MCAD, and ACSL in the failing heart may at least partly depend on AMPK. However, the underlying mechanism of this phenomenon remains unknown.

The present study has further demonstrated that the level of FAO in the failing heart is impaired but that AMPK may take part in adjusting FAO, which may be the main reason for “metabolic remodeling” in the failing heart, being provoked by HF and exacerbating HF in turn. The above effects of *Gs-Rb1* on HF, from the FAO-related enzymes to ATP content, suggest that FAO function may be improved by *Gs-Rb1* in the failing heart. The synergistic studies, along with *Ara A* and *Aicar*, revealed that the FAO effects of *Gs-Rb1* at least partly depended on AMPK activity. However, we predict that other additional mechanisms exist that allow for *Gs-Rb1* maintenance of myocardial FAO rates independent from the AMPK signal. 

## Conclusion

According to the findings of the present study, especially the results in which the effects of *Gs-Rb1* improve HF significantly accompanied by a significant increase in PCr, ADP, ATP, PCr/ATP ratio, free carnitine, malonyl-CoA, both mRNA and activity of Cpt, MCAD, and ACSL, and a significant decrease of the ADP/ATP ratio in left ventricular myocardium, and all those effects were almost abolished by *Ara A* and were not further improved by *Aicar*, we suggest that the capacities of *Gs-Rb1* adjusting myocardial FAO in the failing heart play a key role in its effects on improving HF, and all those may be mediated via activating AMPK pathway. So *Gs-Rb1* could be suspected as one of potential HF drug treatments, but further studies on this particular topic are essential. 
